# Reversible N_2_ binding and photoreduction at dinuclear sites in dynamic metal-organic frameworks

**DOI:** 10.1093/nsr/nwag253

**Published:** 2026-04-30

**Authors:** Anqi Zhang, Zedong Zhang, Danyang Xu, Keke Tu, Xiaocheng Zhou, Zixuan Chen, Yan Xiong, Shuai Yuan, Jing-Lin Zuo

**Affiliations:** State Key Laboratory of Coordination Chemistry, School of Chemistry and Chemical Engineering, Nanjing University, Nanjing 210023, China; State Key Laboratory of Coordination Chemistry, School of Chemistry and Chemical Engineering, Nanjing University, Nanjing 210023, China; State Key Laboratory of Coordination Chemistry, School of Chemistry and Chemical Engineering, Nanjing University, Nanjing 210023, China; Suzhou Key Laboratory of Green Intelligent Manufacturing of New Energy Materials and Devices, Institute of Green Chemistry and Engineering, Nanjing University, Suzhou 215163, China; State Key Laboratory of Analytical Chemistry for Life Science, School of Chemistry and Chemical Engineering, Nanjing University, Nanjing 210023, China; State Key Laboratory of Coordination Chemistry, School of Chemistry and Chemical Engineering, Nanjing University, Nanjing 210023, China; State Key Laboratory of Analytical Chemistry for Life Science, School of Chemistry and Chemical Engineering, Nanjing University, Nanjing 210023, China; State Key Laboratory of Coordination Chemistry, School of Chemistry and Chemical Engineering, Nanjing University, Nanjing 210023, China; Suzhou Key Laboratory of Green Intelligent Manufacturing of New Energy Materials and Devices, Institute of Green Chemistry and Engineering, Nanjing University, Suzhou 215163, China; State Key Laboratory of Coordination Chemistry, School of Chemistry and Chemical Engineering, Nanjing University, Nanjing 210023, China; State Key Laboratory of Coordination Chemistry, School of Chemistry and Chemical Engineering, Nanjing University, Nanjing 210023, China

**Keywords:** coordination chemistry, metal–organic frameworks, crystal engineering, nitrogen fixation, photocatalysis

## Abstract

Direct structural characterization of reactive intermediates in photocatalytic N_2_ reduction is challenging due to their low concentration and fleeting nature, often limiting mechanistic understanding to spectroscopic or computational inference. Herein, we report the crystallographic capture of a coordinatively unsaturated dinuclear intermediate for N_2_ activation, generated through a photoinduced single-crystal-to-single-crystal transformation within a dynamic coordination polymer. The initial structure, NJUZ-Zn, is an active photocatalyst for N_2_ photoreduction and contains a stable [Zn^2+^–(N≡N)^−^–Zn^2+^] site with bridging N_2_^−^ anions. Upon irradiation, N_2_^−^ dissociation was observed by single-crystal X-ray diffraction, yielding the unsaturated [Zn^2+^···Zn^+^] center. Raman spectroscopy and isotope-tracking experiments demonstrated the reversible exchange of ^14^N_2_ and ^15^N_2_ at the bimetallic site, confirming dynamic interconversion between saturated and unsaturated states during photocatalysis. Together, these results establish a photocatalytic mechanism in which dissociation of bridging N_2_^−^ generates reactive bimetallic intermediates that continuously capture and activate external N_2_ to produce ammonia under ambient conditions. This work provides unambiguous structural evidence of a key intermediate in an active photocatalyst for N_2_ fixation, offering a blueprint for understanding the reaction pathway and guiding the rational design of cooperative metal sites for sustainable catalysis.

## Teaser

This study presents the crystallographic capture of a coordinatively unsaturated dinuclear intermediate in photocatalytic N₂ reduction, revealing dynamic interconversion and N₂ activation for sustainable ammonia production under ambient conditions.

## INTRODUCTION

The conversion of dinitrogen (N_2_) into ammonia is fundamental to both industry and life. However, owing to its nonpolar nature, high bond dissociation energy (941 kJ mol^−1^), large gap between the highest occupied molecular orbital (HOMO) and the lowest unoccupied molecular orbital (LUMO) (10.82 eV), and high-lying 1*π*_g_ LUMO (−7.91 eV) [1,2], N_2_ exhibits exceptional chemical inertness, making its activation a fundamental challenge in catalysis [3]. The industrial Haber-Bosch process, the primary method for nitrogen fixation, consumes significant energy due to high temperature and pressure requirements. In contrast, biological nitrogen fixation, which is mediated by nitrogenases, occurs under ambient conditions through multi-electron transfer facilitated by complex metalloclusters.

Nitrogenases are categorized based on their active site composition into Mo-, V-, and Fe-nitrogenases, each exhibiting distinct catalytic efficiencies and mechanistic features. Mo-nitrogenase, which contains an FeMo-cofactor ([MoFe_7_S_9_C]), is the most efficient and well-characterized system, enabling N_2_ activation at a reduced Fe site via sequential protonation to yield NH_3_ [4–10]. V-nitrogenase, incorporating the FeV-cofactor ([VFe_7_S_8_(CO_3_)C]), follows a similar mechanism but exhibits lower catalytic efficiency and occasionally produces hydrazine (N_2_H_4_) as an intermediate [11–13]. Fe-nitrogenase, which uses an FeFe-cofactor ([FeFe_7_S_8_]), remains the least understood but is presumed to follow a comparable catalytic pathway with reduced turnover rates [14]. The reduction of N_2_ at nitrogenase metalloclusters occurs through distinct coordination modes, broadly categorized as terminal and bridging coordination [15–21]. In the terminal mode, N_2_ binds to a single metal center via one nitrogen atom, forming an M–N≡N structure that facilitates *π*-back bonding, thereby weakening the N≡N bond to promote stepwise protonation and reduction. In contrast, the bridging mode involves N_2_ coordination across two or more cooperative metal centers (M–N≡N–M) [12, 22, 23], offering enhanced stability and improved nitrogen activation capabilities.

Despite extensive research, reproducing the efficiency of nitrogenase in artificial systems remains a formidable challenge [24–26]. Considerable efforts have focused on the development of metal-based catalysts that mimic nitrogenase function, particularly low-valence transition metal complexes such as those based on Ru and Mo, which have demonstrated promising N_2_ fixation and activation capabilities [27–32]. Photochemical nitrogen activation has also been explored, achieving N≡N bond cleavage under light irradiation [33]. Additionally, metal-sulfur clusters, similar to nitrogenase cofactors, have shown enhanced catalytic activity through multi-electron transfer mechanisms [34–38]. However, many homogenous N_2_ reduction catalysts often suffer from limited stability and low efficiency under ambient conditions, restricting their practical applications. On the other hand, the heterogeneous systems lack precise structural evidence for key intermediates, thus hindering mechanistic understanding and rational catalyst design.

Porous coordination polymers or metal‒organic frameworks (MOFs) offer an attractive solution to these limitations [39,40]. Their tunable structure enables the creation of dinuclear metal sites to mimic enzymatic active centers for N_2_ fixation, while their crystalline nature and well-defined structures provide an ideal matrix for monitoring reaction intermediates and pathways by crystallography [41–48]. Nevertheless, conventional MOF-based catalysts often exhibit limited N_2_ activation capabilities under mild conditions. We previously developed a new strategy for nitrogen fixation by stabilizing dinitrogen anions as bridging ligands between dinuclear cooperative metal centers of coordination polymers, leading to efficient photocatalytic nitrogen activation at ambient temperature and pressure. This type of coordination polymer, NJUZ-Zn, has been demonstrated as an efficient nitrogen fixation photocatalyst under ambient conditions [49], but its active intermediate and detailed catalytic mechanism remain unresolved.

In this work, we extend the platform to a cadmium analog (NJUZ-Cd) and isolate the key intermediate via photocrystallography, providing direct structural insight into the catalytic mechanism. Single-crystal X-ray diffraction (SC-XRD) reveals that the [Zn^2+^–(N≡N)–Zn^2+^] site in NJUZ-Zn undergoes light-induced N_2_^−^ dissociation, generating the coordinatively unsaturated [Zn^2+^···Zn^+^] center in NJUZ-Int. X-ray absorption spectroscopy (XAS) further corroborates the partial reduction of Zn^2+^ into Zn^+^, accompanied by a decrease in coordination number from 6 to 5. Complementary Raman spectroscopy and isotope-tracking experiments reveal the reversible exchange of ^14^N_2_ and ^15^N_2_ at the dinuclear site, confirming dynamic cycling between saturated and unsaturated states under photocatalytic conditions. This dynamic process sustains the continuous generation of reactive [Zn^2+^···Zn^+^] intermediates that capture and activate N_2_, generating N_2_^−^ species that subsequently convert into ammonia under ambient conditions. Our findings establish a light-driven nitrogen fixation mechanism mediated by dinitrogen exchange at dinuclear metal centers, highlighting a promising strategy for efficient N_2_ conversion under ambient conditions and advancing the development of sustainable nitrogen fixation technologies.

## RESULTS AND DISCUSSION

### Structural design and synthesis of NJUZ-M (M = Zn, Cd) photocatalysts

**Figure 1.**
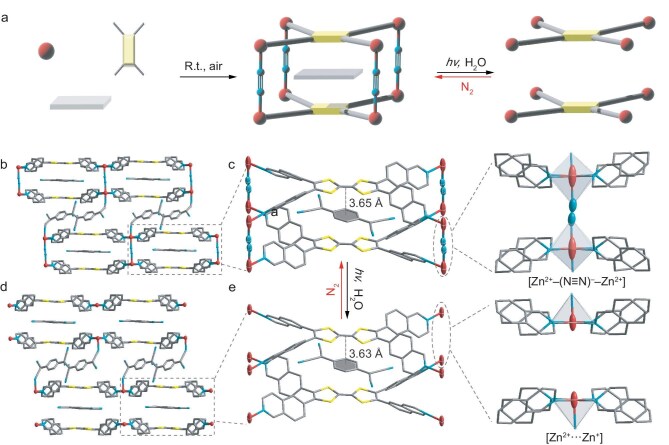
Structural design and crystal structure of NJUZ-M. (a) Dynamic coordination polymers for photocatalytic nitrogen fixation under mild conditions. (b) Ball-and-stick view of the 3D framework of NJUZ-Cd with TCNQ guest molecules, viewed along the *a* axis. (c) A TCNQ molecule hosted in the cavity of the [(L)_2_(Cd_2_N_2_)_4_] structure. Selected bond lengths and angles: N–N 1.110(8) Å. (d) Ball-and-stick view of the 3D framework of NJUZ-Int with TCNQ guest molecules, viewed along the *a* axis. (e) A TCNQ molecule hosted in the cavity of the [(L)_2_(Zn_2_)_4_] structure. Selected bond lengths and angles: Zn–Zn 5.876 Å.

The coordination polymer NJUZ-Cd was synthesized by the reaction of L and lithium tetracyanoquinodimethane [Li(TCNQ)] ligands with cadmium nitrate in a mixed solvent of *N,N*-dimethylformamide, CHCl_3_, and CH_3_OH at room temperature under an air atmosphere (Fig. 1a). SC-XRD analysis reveals that NJUZ-Cd is a (2,4,6) 3-connected three-dimensional (3D) network structure (Fig. 1b). Each asymmetric unit is composed of one independent Cd^2+^ center, half of N_2_, one L ligand, half of the TCNQ–TCNQ dimer and half of the TCNQ moiety (Fig. S2a and Tables S1–S3). Its secondary building units (SBUs) consist of [Cd_2_N_2_] pillars formed by two cadmium centers bridged by an end-on N_2_^−^ anion (Fig. 1c). Four SBUs coordinate with two L ligands to form a cavity [(L)_2_(Cd_2_N_2_)_4_], which encapsulates a TCNQ guest molecule (Fig. S3). The TCNQ molecule and tetrathiafulvalene moiety from L form *π*–*π* stacking with a distance of 3.652 Å (Fig. 1c). The cavities of {(L)_2_[Cd_2_(N_2_)]_4_} are expanded into a 2D layered structure through SBU interactions in the *bc* plane (Fig. S2b and c). Adjacent 2D layers are further interconnected through TCNQ–TCNQ dimer ligands, ultimately forming a 3D framework (Fig. S2d).

**Figure 2.**
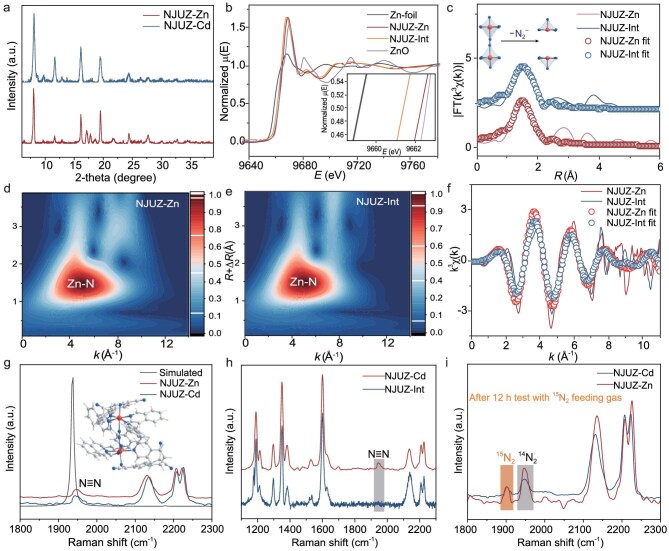
Structural characterization diagram of NJUZ-M (M = Zn, Cd). (a) XRD spectra of NJUZ-M (M = Zn, Cd). (b) The normalized X-ray absorption near-edge spectra at the Zn *K*-edge. (c) Zn *K*-edge *k*^3^*χ*(*k*) functions of all the samples. (d and e) Wavelet Transformation for the *k*^3^-weighted EXAFS signal of Zn samples. (f) The *k*^3^-weighted Fourier transform extended X-ray absorption fine structure spectra (EXAFS) in r-space. (g) SERS spectra of NJUZ–M (M = Zn, Cd) grown in air. The inset shows the simulated Raman spectrum of the Zn–N_2_–Zn structure, calculated at level 1 using the DFT method, compared with the experimental result. (h) SERS spectra of NJUZ-Cd and NJUZ-Int. (i) SERS spectra of NJUZ-M (M = Zn, Cd) after the photocatalytic reaction in ^15^N_2_ for 12 h.

The phase purity of NJUZ-Cd was confirmed by powder XRD measurements, with the diffraction peak positions consistent with those of NJUZ-Zn, indicating potential isomorphism with NJUZ-Zn (Fig. 2a). Infrared spectroscopy revealed two split peaks at 2100 cm^−1^, corresponding to the cyanide stretching vibrations of TCNQ (Fig. S6). Scanning electron microscopy and energy-dispersive X-ray spectroscopy mapping revealed a uniform distribution of metal and nitrogen throughout the material (Figs S7 and S8). Solid-state electron paramagnetic resonance (EPR) spectroscopy revealed characteristic double-splitting differential signals in the X-band region at room temperature (Fig. S9), with a calculated *g* value of 2.003, typical of organic unpaired electrons. A weak half-field signal was also observed, supporting a triplet-like (*S* = 1) weakly coupled two-spin state associated with the N_2_-bridged dinuclear site rather than an isolated *S* = 1/2 radical [49]. To assess a possible ligand-based origin, we compared the EPR spectra of the neutral ligand L, Li-TCNQ, NJUZ-Int, and the parent framework (Fig. S9a–c). The neutral ligand L is essentially EPR silent, and although Li-TCNQ is EPR active, its spectral profile differs markedly from that of NJUZ-Cd. In addition, NJUZ-Int, which lacks the bridging dinitrogen unit, shows a strongly attenuated signal. These results indicate that the characteristic EPR response is closely associated with the N_2_-bridged dinuclear site, rather than arising simply from the organic linker or TCNQ alone. ENDOR measurements revealed ^1^H hyperfine coupling, which is not inconsistent with this assignment, because the observed proton coupling can arise from partial spin delocalization onto the TCNQ/framework environment, and possibly nearby adsorbed water or hydrogen-bonding environments, rather than requiring that the radical core itself contains hydrogen. Quantitative EPR analysis based on double integration of the X-band signal, calibrated against TEMPOL/KBr solid standards measured under identical conditions, gave an apparent spin concentration of 2.07 × 10^19^ spins g^−1^, corresponding to 3.89 × 10^−2^ mol radical mol^−1^ Zn. We therefore describe this value as a calibrated apparent radical amount derived from EPR rather than a strict stoichiometric electron count (Fig. S9d and e). This finding is in good agreement with the above analysis and confirms the existence of the dinitrogen anion. The high porosity and ultrathin configuration of these nanosheets lead to high nitrogen adsorption at room temperature (Fig. S10a), resulting in open channels and accessible active sites. Moreover, diffuse reflectance spectroscopy demonstrated that NJUZ-Cd has strong light absorption in the ultraviolet and visible regions (Fig. S10b), which is attributed to metal-to-ligand charge transfer or intermolecular charge transfer between tetrathiafulvalene (TTF) and TCNQ molecules. All these advantageous structural features and physicochemical properties of NJUZ-Cd hold tremendous possibilities for photocatalytic nitrogen fixation.

To further verify the presence of bridging N_2_^−^ anions in NJUZ-Cd, surface-enhanced Raman scattering (SERS) spectroscopy was employed. Density functional theory (DFT) (level 1: B3LYP-D3) predicted that the N≡N stretching vibration in the M–N_2_–M structure is ∼1937 cm^−1^, whereas the Cd–CN–Cd model was ruled out because of the absence of characteristic peaks in the 1900–2100 cm^−1^ region (Fig. S12). In the Raman spectrum, the vibration peak observed at a lower wavenumber of ∼1945 cm^−1^ should be attributed to the instantaneous dipole moment of the symmetric dinitrogen anion (Fig. 2g), which is consistent with previously reported dinitrogen anion complexes.

### Capture of the [Zn^2+^···Zn^+^] intermediate

Taking advantage of the precise crystal structure and NJUZ-M (M = Zn, Cd), we attempted to capture the [Zn^2+^···Zn^+^] intermediate during photocatalytic N_2_ reduction by single-crystal-to-single-crystal transformation. Upon irradiation of NJUZ-Zn under 300 W Xe lamp light for 2 h, the unsaturated intermediate NJUZ-Int was obtained, which lacks the bridging dinitrogen anions present in NJUZ-Cd and NJUZ-Zn (Figs 1c and S4, and Table S4). SC-XRD reveals a distinct change in the Zn coordination environment. In NJUZ-Zn, each Zn center adopts a six-coordinate octahedral geometry, with four equatorial N-donors from the L ligands, one axial N from the dinitrogen bridge, and a second axial N from the TCNQ moiety. Upon irradiation, dissociation of the bridging (N≡N)^−^ ligand occurs, yielding NJUZ-Int in which the Zn centers adopt a five-coordinate square-pyramidal geometry. The equatorial plane remains occupied by four nitrogen donors from the L ligands, while only a single axial coordination ligand is retained, leaving the second axial position vacant. This generates the coordinatively unsaturated [Zn^2+^···Zn^+^] intermediate, in which the vacant site can serve as an entry point for new N_2_ molecules.

Notably, the overall lattice framework is preserved during this single-crystal-to-single-crystal transformation. The unit cell volume and lattice parameters of NJUZ-Zn and NJUZ-Int remain nearly identical (Table S1), and the Zn···Zn separations are unchanged, highlighting the robustness of the structural scaffold in accommodating local coordination changes. The strong donor-acceptor-donor (D-A-D) interactions between the tetrathiafulvalene moiety of the L ligands and the *π*-accepting TCNQ units play a crucial role in stabilizing the unsaturated geometry of NJUZ-Int and preventing collapse of the framework (Fig. S5). The maintenance of crystallinity, coupled with the local conversion from octahedral to square-pyramidal coordination, underscores the dynamic nature of this coordination polymer.

To elucidate the changes in oxidation state and coordination environment of Zn centers in NJUZ-Zn and NJUZ-Int, we performed XAS analysis, including X-ray absorption near-edge structure (XANES) and extended X-ray absorption fine structure (EXAFS). As shown in Fig. 2b, the Zn *K*-edge XANES spectra of both NJUZ-Zn and NJUZ-Int exhibit absorption edge energies that lie between those of Zn foil (Zn(0)) and ZnO (Zn(II)), suggesting that Zn exists in an oxidized state (Zn*^δ^*^+^, where 0 < *δ*_Int_ < *δ*_Zn_ ≤ 2). While the oxidation state of Zn in NJUZ-Zn is close to +2, the oxidation state of Zn in NJUZ-Int, obtained after illumination, is lower than that of Zn in NJUZ-Zn, which is consistent with the partial reduction of Zn^2+^ to Zn^+^ upon (N≡N)^−^ leaving [50–53]. The Fourier transform curves of Zn *K*-edge EXAFS *k*^3^*χ*(*k*) oscillatory functions for both NJUZ-Zn and NJUZ-Int (Fig. 2c) reveal a prominent peak at approximately 1.49 Å, characteristic of Zn−N coordination (Fig. S11), with a bond length of 2.15 Å (Table S4), which corresponds to the bond length observed in single crystal data. Wavelet transform analysis (Fig. 2d and e) further demonstrates a peak at *R* ∼1.49 Å/*k* ∼4.2 Å^−1^ (Fig. S12), which is exclusively attributed to Zn–N coordination (Fig. S11). Quantitative EXAFS fitting reveals subtle yet significant differences in local coordination: an average coordination number of 5.7 for NJUZ-Zn, consistent with octahedral Zn^2+^ centers, and 5.1 for NJUZ-Int, consistent with a square-pyramidal environment (Fig. 2f and Table S5). These spectroscopic results align closely with the single-crystal structural data, confirming that illumination triggers a transformation from six-coordinated Zn^2+^ sites in NJUZ-Zn to five-coordinated Zn^2+^/Zn^+^ centers in NJUZ-Int. Together, these results provide direct structural evidence for the reactive [Zn^2+^···Zn^+^] intermediate for the dynamic N_2_ binding and activation process.

### Monitoring reversible N_2_ binding by Raman spectroscopy and isotope exchange

Raman spectroscopy was employed to probe the dynamic N_2_ coordination within NJUZ-M (M = Zn, Cd). Pristine NJUZ-M displays a characteristic band at ∼1945 cm^−1^, assigned to the ^14^N≡^14^N stretching mode of the bridging dinitrogen anion at the saturated [M^2+^–(N≡N)^−^–M^2+^] sites (Fig. 2c). In contrast, NJUZ-Int exhibits nearly identical spectral features except for the disappearance of this 1945 cm^−1^ band, consistent with loss of the bridging N_2_^−^ anions. Exposure of NJUZ-M (M = Zn, Cd) to a pure ^15^N_2_ atmosphere for 12 h generated a new band at 1905 cm^−1^ (Fig. 2i), attributable to the ^15^N≡^15^N stretching vibration. The observed redshift of ∼40 cm^−1^ relative to the ^14^N≡^14^N signal is consistent with Hooke’s law calculations, which predict a value of ∼1920 cm^−1^ for the ^15^N≡^15^N stretch.

**Figure 3.**
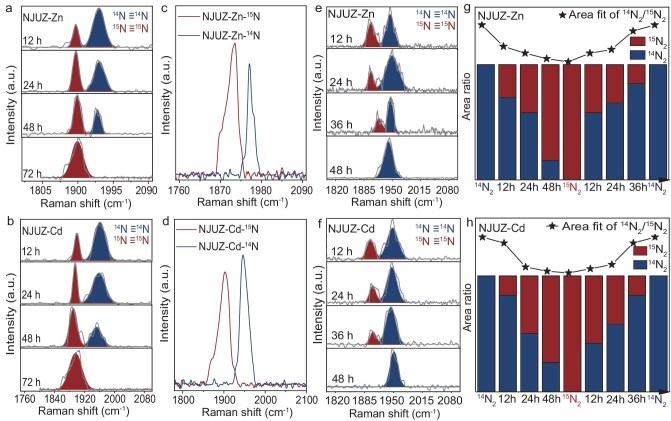
Raman spectra of NJUZ-M (M = Zn, Cd) at different photocatalytic reaction times (12, 24, 36, 48 and 72 h) in a ^15^N_2_ atmosphere: (a) NJUZ-Zn, (b) NJUZ-Cd. Raman spectra of single crystals grown in ^15^N_2_ and ^14^N_2_ atmospheres: (c) NJUZ-Zn, (d) NJUZ-Cd. The Raman spectrum was obtained by using NJUZ-M-^15^N_2_ to perform a photocatalytic reaction in a ^14^N_2_ atmosphere: (e) NJUZ-Zn, (f) NJUZ-Cd. The ratio of the Raman peak fitting areas of ^14^N_2_ and ^15^N_2_ of NJUZ-M under different reaction atmospheres and for different reaction times: (g) NJUZ-Zn, (h) NJUZ-Cd.

Time-resolved isotope exchange experiments highlight the dynamic cycling of nitrogen species at the dinuclear sites. When NJUZ-M-^14^N_2_ was irradiated in a ^15^N_2_ atmosphere, the 1905 cm^−1^ band progressively grew in intensity with reaction time, while the 1945 cm^−1^ band diminished. After 48 h, the ^15^N≡^15^N peak dominated the spectrum. By 72 h, the ^14^N≡^14^N signal completely disappeared, confirming nearly quantitative isotopic replacement (Fig. 3a and b, Figs S14–S20). These results demonstrate that the saturated [M^2+^–(^14^N≡^14^N)^−^−M^2+^] sites continuously dissociate to form the unsaturated [M^2+^···M^+^] intermediates, which in turn bind ^15^N_2_ molecules from the environment. Importantly, isotope exchange conducted in the dark showed no spectral changes, confirming that this reversible binding and exchange process is photoactivated (Fig. S13).

Additional validation was obtained by synthesizing NJUZ-M directly under a ^15^N_2_ atmosphere. The resulting material exhibited a dominant 1905 cm^−1^ peak, whereas NJUZ-M prepared under ^14^N_2_ displayed only the 1945 cm^−1^ feature (Fig. 3c and d, Figs S21 and S22). To confirm the reversibility of this process, NJUZ-M-^15^N_2_ pre-equilibrated for 48 h was subsequently exposed to ^14^N_2_. Over time, the ^15^N≡^15^N signal weakened while the ^14^N≡^14^N signal reappeared, completing a full exchange cycle (Fig. 3e and f, Figs S23–S26). The corresponding Raman peak area ratios (Fig. 3g and h, Figs S25 and S26) further confirmed the quantitative conversion between [M^2+^–(^14^N≡^14^N)^−^–M^2+^] and [M^2+^–(^15^N≡^15^N)^−^–M^2+^]. XRD analysis after the reaction revealed that NJUZ-M maintains its structural integrity during the repeated exchange of (N≡N)^−^ (Fig. 27), demonstrating the stability of the coordination polymer during the isotopic exchange cycles.

### Photocatalytic performance and proposed reaction pathways

**Figure 4.**
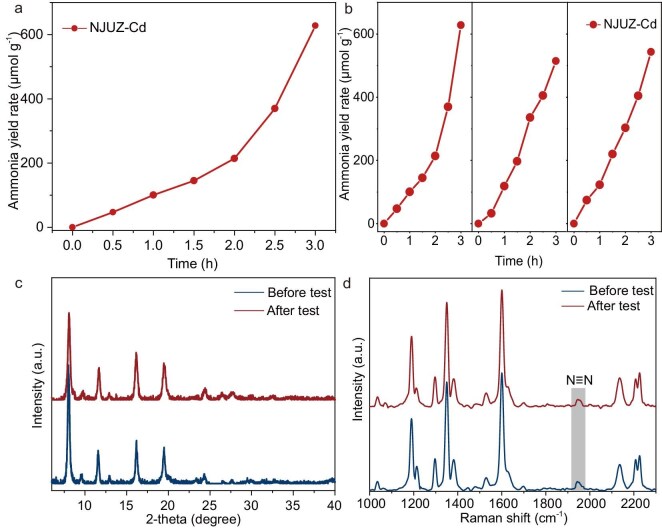
(a) Total ammonia yield rates of NJUZ-Cd in deionized water saturated by N_2_ and (b) 10 h long-term stability test for the NRR with N_2_ flow. Each experiment was repeated independently at least three times with similar results for the same sample, and representative data are shown. (c) XRD spectra, and (d) SERS spectra of NJUZ-Cd before and after the cyclic reaction.

The photocatalytic nitrogen reduction reaction (NRR) activity of NJUZ-Cd was evaluated in deionized water under irradiation with a 300 W Xe lamp using purified N_2_ as the feed gas without the sacrificial donor. Ammonia formation was quantified spectrophotometrically by the indophenol blue method. In this system, water serves not only as the proton source but also as the ultimate sacrificial electron donor through hole-consuming oxidation, while the photogenerated electrons are delivered to the [Cd–N≡N–Cd] active sites through the donor–acceptor framework to drive dinitrogen activation and reduction. As shown in Fig. 4a, the ammonia produced by the NJUZ-Cd photocatalyst with purified N_2_ flow as feeding gas is 134 μmol g^−1^ h^−1^. The catalytic stability of the NJUZ-Cd photocatalyst was measured with three cycles (Fig. 4b), and the ammonia yield rate is consistent with each other for the three dependent tests, which confirms that NJUZ-Cd has very high photocatalytic stability. The structural stability of NJUZ-Cd was also measured via XRD and Raman, as shown in Fig. 4c and d. XRD revealed that the crystalline structure of NJUZ-Cd remains almost unchanged without structural degradation after photocatalytic cycling. The Raman intensity of NJUZ-Cd remained stable before and after the NRR, indicating that the [Cd–N≡N–Cd] site is stable. Notably, as demonstrated in Fig. S28, NJUZ-M-^15^N_2_ undergoes a photocatalytic reaction in unpurified air as the feed gas, and atmospheric N_2_ can still be activated by [M^2+^···M^+^]. Furthermore, the photocatalytic nitrogen reduction activities of NJUZ-Zn and NJUZ-Cd were compared. Under ambient conditions, the ammonia yield of NJUZ-Zn was 140 μmol g^−1^ h^−1^, and both materials exhibited comparable nitrogen reduction activities (Fig. S29 and Table S6). The slight difference in activity may stem from the difference in atomic mass between Zn and Cd. Both Zn^2+^ and Cd^2+^ have closed-shell *d*^10^ electronic configurations, resulting in minimal difference in the intrinsic electronic structure of the active sites. This finding indicates that the NJUZ-M photocatalyst has a high tolerance toward an ambient atmosphere, suggesting its potential for large-scale and cost-effective nitrogen fixation applications.

**Figure 5.**
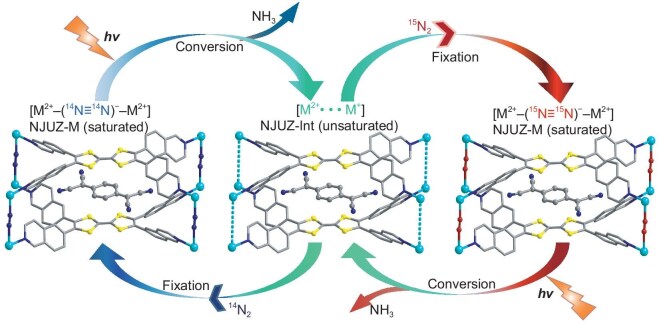
Scheme of the cycling process of NJUZ-M (M = Zn, Cd) during the photocatalytic NRR process.

Based on the single-crystal structural evidence and complementary spectroscopic analyses, we propose a dynamic photocatalytic cycling process for NJUZ-M (M = Zn, Cd) during photocatalytic NRR (Fig. 5). Although coordination of N_2_ to closed-shell d^10^ metal ions such as Zn^2+^ and Cd^2+^ is unusual, in the present system this behavior can be rationalized by the combined structural and electronic features of the framework. Specifically, the dinuclear metal site provides a confined bridging pocket for N_2_ coordination, while the donor–acceptor framework promotes charge redistribution and stabilizes the N_2_-bound state, thereby enabling binding and activation of a bridging dinitrogen species at the M_2_ site. The as-synthesized NJUZ-M consists of a bridging (N≡N)^−^ anion coordinated between two metal centers, forming a stable [M^2+^–(N≡N)^−^–M^2+^] unit. Upon photoexcitation, the bridging dinitrogen anion dissociates from the metal centers, generating coordinatively unsaturated [M^2+^···M^+^] intermediates. SC-XRD directly captured this transformation in NJUZ-Int, revealing the conversion of octahedral Zn^2+^ sites into pentacoordinate square-pyramidal Zn^2+^/Zn^+^ centers. The released (N≡N)^−^ species is expected to undergo protonation under reaction conditions, which may subsequently lead to disproportionation pathways yielding NH_3_ and N_2_. Although the precise downstream chemistry of the free (N≡N)^−^ cannot yet be resolved, the structural evidence establishes that the unsaturated [M^2+^···M^+^] intermediates play a central role. These reactive sites can readily capture new N_2_ molecules from the atmosphere, regenerating the saturated [M^2+^–(N≡N)^−^–M^2+^] units via one-electron transfer. Thus, the photocatalytic cycle is sustained through reversible interconversion between saturated dinuclear N_2_-bridged [M^2+^–(N≡N)^−^–M^2+^] complexes and unsaturated [M^2+^···M^+^] intermediates, enabling continuous N_2_ binding, activation, and turnover under mild conditions.

## CONCLUSION

In summary, we have provided direct crystallographic evidence of a key intermediate and tracked the dynamic N_2_ coordination cycle that drives photocatalytic nitrogen reduction in dinuclear coordination polymers. Through a photoinduced single-crystal-to-single-crystal transformation, we captured the unsaturated [Zn^2+^···Zn^+^] intermediate (NJUZ-Int) that underpins N_2_ activation. Complementary Raman and isotope-exchange studies further revealed the reversible interconversion between saturated [M^2+^–(N≡N)^−^–M^2+^] and unsaturated [M^2+^···M^+^] sites. This dynamic binding mode sustains continuous N_2_ uptake and turnover, enabling efficient ammonia production under ambient conditions. These findings not only illuminate the reaction pathway in a highly active nitrogen fixation system but also establish dynamic coordination polymers as a powerful platform for trapping and characterizing active intermediates, thereby paving the way for the rational design of next-generation heterogeneous catalysts for sustainable activation of inert molecules.

## METHODS

### Synthesis of NJUZ-M (M = Zn, Cd)

NJUZ-M was prepared based on L and Li(TCNQ). A solution of Zn(NO_3_)_2_·6H_2_O or Cd(NO_3_)_2_·4H_2_O (0.04 mmol) in CH_3_OH was allowed to diffuse into the solution of L (0.01 mmol) and Li(TCNQ) (0.02 mmol) in CHCl_3_ and dimethylformamide at atmospheric pressure and room temperature. This produced shining dark crystals suitable for XRD analysis after they were kept for ∼1 week on the wall of a test tube. Yield: 57% based on L. Elemental analysis of NJUZ-M—calculated: C 60.10, N 12.64, H 2.55%; found: C 58.43, N 11.42, H 3.04%. Unless specified otherwise, this synthesis method was also used to prepare bulk samples for the following characterizations and photocatalysis experiments. Isotope-labeled ^15^N-NJUZ-M was synthesized similarly to NJUZ-M, except that all steps used Schlenk technology under a ^15^N_2_ environment. The mixture was left for 2 weeks at room temperature and dark red crystals were obtained. Yield: 32% based on L.

### Synthesis of NJUZ-Int

Under Ar gas, NJUZ-Zn was irradiated in water for 2 h to obtain NJUZ-Int.

### Structural analysis of NJUZ-Cd single crystals

The SC-XRD study shows that each asymmetric unit of NJUZ-Cd contains one independent Cd center, half of N_2_, one L ligand, half of the TCNQ-TCNQ dimer, and half of the TCNQ moiety (Fig. 1b). As shown in Fig. 1b, the central Cd (II) ion adopts a distorted octahedral coordination environment, which is defined by four nitrogen donors from four L ligands in the equatorial plane and two nitrogen donors from dinitrogen and the TCNQ-TCNQ dimer. In a [(L)_2_(Cd_2_N_2_)_4_] cavity, a TCNQ molecule is sandwiched between two TTF units with an average *π*···*π* stacking distance of 3.652 Å, while the hydrogen atoms on the eight isoquinoline groups point toward the N atoms of TCNQ with C–H···N distances ranging from 2.667 to 3.022 Å (Fig. S2). It is worth noting that TCNQ acts as a μ-dimer and a single radical molecule in the NJUZ-Cd crystal lattice. The central C=C bond length of the TTF core is 1.323 Å (Fig. S2), suggesting that the TTF moiety is neutral. The scarcity of other supplementary charged species in the crystal channel confirms the distribution of the valences in the asymmetric unit. In brief, one positive divalent Cd^2+^ ion completely corresponds to half of a negative divalent bridging dimer, half of a guest radical and half of an unidentified dinitrogen anion.

## Supplementary Material

nwag253_Supplemental_Files

## Data Availability

All experimental details and data supporting the findings of this study are available within the paper and its Supplementary Information. The data are also available from the Corresponding author upon reasonable request. Crystallographic data for the NJUZ-Cd and NJUZ-Int structures have been deposited at the Cambridge Crystallographic Data Centre, under deposition numbers CCDC 2429955 and 2488818. Copies of the data can be obtained free of charge via https://www.ccdc.cam.ac.uk/structures/.

## References

[1] Jia HP, Quadrelli EA. Mechanistic aspects of dinitrogen cleavage and hydrogenation to produce ammonia in catalysis and organometallic chemistry: relevance of metal hydride bonds and dihydrogen. Chem Soc Rev 2014; 43: 547–64. 10.1039/C3CS60206K 24108246

[2] Hinchliffe A . Chemical modelling: applications and theory. Cambridge: Royal Society of Chemistry, 2008, 17–62.

[3] Himmel HJ, Reiher M. Intrinsic dinitrogen activation at bare metal atoms. Angew Chem Int Ed 2006; 45: 6264–88. 10.1002/anie.200502892 16983726

[4] Lancaster KM, Roemelt M, Ettenhuber P et al. X–ray emission spectroscopy evidences a central carbon in the nitrogenase iron–molybdenum cofactor. Science 2011; 334: 974–7. 10.1126/science.1206445 22096198 PMC3800678

[5] Georgiadis MM, Komiya H, Chakrabarti P et al. Crystallographic structure of the nitrogenase iron protein from azotobacter vinelandii. Science 1992; 257: 1653–9. 10.1126/science.1529353 1529353

[6] Spatzal T, Aksoyoglu M, Zhang L et al. Evidence for interstitial carbon in nitrogenase FeMo cofactor. Science 2011; 334: 940. 10.1126/science.1214025 22096190 PMC3268367

[7] Wiig JA, Hu Y, Lee CC et al. Radical SAM–dependent carbon insertion into the nitrogenase M–cluster. Science 2012; 337: 1672–5. 10.1126/science.1224603 23019652 PMC3836454

[8] Schindelin H, Kisker C, Schlessman JL et al. Structure of ADP–AlF_4_ stabilized nitrogenase complex and its implications for signal transduction. Nature 1997; 387: 370–6. 10.1038/387370a0 9163420

[9] Tezcan FA, Kaiser JT, Mustafi D et al. Nitrogenase complexes: multiple docking sites for a nucleotide switch protein. Science 2005; 309: 1377–80. 10.1126/science.1115653 16123301

[10] Danyal K, Dean DR, Hoffman BM et al. Electron transfer within nitrogenase: evidence for a deficit–spending mechanism. Biochem 2011; 50: 9255–63. 10.1021/bi201003a 21939270 PMC3202676

[11] Sippel D, Einsle O. The structure of vanadium nitrogenase reveals an unusual bridging ligand. Nat Chem Biol 2017; 13: 956–60. 10.1038/nchembio.2428 28692069 PMC5563456

[12] Sippel D, Rohde M, Netzer J et al. A bound reaction intermediate sheds light on the mechanism of nitrogenase. Science 2018; 359: 1484–9. 10.1126/science.aar2765 29599235

[13] Eady RR . Structure–function relationships of alternative nitrogenases. Chem Rev 1996; 96: 3013–30. 10.1021/cr950057h 11848850

[14] Krahn E, Weiss BJR, Kröckel M et al. The Fe–only nitrogenase from Rhodobacter capsulatus: identification of the cofactor, an unusual, high–nuclearity iron–sulfur cluster, by Fe K–edge EXAFS and ^57^Fe Mössbauer spectroscopy. J Biol Inorg Chem 2002; 7: 37–45. 10.1007/s007750100263 11862539

[15] Deng H, Hoffmann R. How N_2_ might be activated by the FeMo–Cofactor in nitrogenase. Angew Chem Int Ed Engl 1993; 32: 1062–5. 10.1002/anie.199310621

[16] Kästner J, Blöchl PE. Ammonia production at the FeMo cofactor of nitrogenase: results from density functional theory. J Am Chem Soc 2007; 129: 2998–3006. 10.1021/ja068618h 17309262

[17] Varley JB, Wang Y, Chan K et al. Mechanistic insights into nitrogen fixation by nitrogenase enzymes. Phys Chem Chem Phys 2015; 17: 29541–7. 10.1039/C5CP04034E 26366854

[18] Siegbahn PEM . Is there computational support for an unprotonated carbon in the E_4_ state of nitrogenase? J Comput Chem 2018; 39: 743–7. 10.1002/jcc.25145 29265384

[19] Dance I . How feasible is the reversible S–dissociation mechanism for the activation of FeMo–co, the catalytic site of nitrogenase? Dalton Trans 2019; 48: 1251–62. 10.1039/C8DT04531C 30607401

[20] Cao L, Ryde U. Extremely large differences in DFT energies for nitrogenase models. Phys Chem Chem Phys 2019; 21: 2480–8. 10.1039/C8CP06930A 30652711

[21] Li D, Zan LX, Chen SM et al. Direct conversion of N_2_ and O_2_: status, challenge nd perspective. Natl Sci Rev 2022; 9: nwac042. 10.1093/nsr/nwac042 36726637 PMC9885431

[22] Seefeldt LC, Yang ZY, Lukoyanov DA et al. Reduction of substrates by nitrogenases. Chem Rev 2020; 120: 5082–106. 10.1021/acs.chemrev.9b00556 32176472 PMC7703680

[23] Spatzal T, Perez KA, Einsle O et al. Ligand binding to the FeMo–cofactor: structures of CO–bound and reactivated nitrogenase. Science 2014; 345: 1620–3. 10.1126/science.1256679 25258081 PMC4205161

[24] MacKay BA, Fryzuk MD. Dinitrogen coordination chemistry: on the biomimetic borderlands. Chem Rev 2004; 104: 385–402. 10.1021/cr020610c 14871129

[25] Hoffman BM, Lukoyanov D, Yang ZY et al. Mechanism of nitrogen fixation by nitrogenase: the next stage. Chem Rev 2014; 114: 4041–62. 10.1021/cr400641x 24467365 PMC4012840

[26] Zhang Y, Zuo JL, Zhou HC et al. Rearrangement of symmetrical dicubane clusters into topological analogues of the P cluster of nitrogenase: nature’s choice? J Am Chem Soc 2002; 124: 14292–3. 10.1021/ja0279702 12452688

[27] Allen AD, Senoff CV. Nitrogen open tammine ruthenium (II) complexes. Chem Commun (London) 1965; 24: 621–2. 10.1039/c19650000621

[28] Yandulov DV, Schrock RR. Catalytic reduction of dinitrogen to ammonia at a single molybdenum center. Science 2003; 301: 76–8. 10.1126/science.1085326 12843387

[29] Curley JJ, Cook TR, Reece SY et al. Shining light on dinitrogen cleavage: structural features, redox chemistry, and photochemistry of the key intermediate bridging dinitrogen complex. J Am Chem Soc 2008; 130: 9394–405. 10.1021/ja8002638 18576632

[30] Fajardo JJR, Peters JC. Catalytic nitrogen–to–ammonia conversion by osmium and ruthenium complexes. J Am Chem Soc 2017; 139: 16105–8. 10.1021/jacs.7b10204 29073760 PMC6019285

[31] Kang W, Lee CC, Jasniewski AJ et al. Structural evidence for a dynamic metallocofactor during N_2_ reduction by Mo–nitrogenase. Science 2020; 368: 1381–5. 10.1126/science.aaz6748 32554596 PMC8410457

[32] Arashiba K, Miyake Y, Nishibayashi Y. A molybdenum complex bearing PNP–type pincer ligands leads to the catalytic reduction of dinitrogen into ammonia. Nat Chem 2011; 3: 120–5. 10.1038/nchem.906 21258384

[33] Meng SL, Li XB, Tung CH et al. Nitrogenase inspired artificial photosynthetic nitrogen fixation. Chem 2021; 7: 1431–50. 10.1016/j.chempr.2020.11.002

[34] Chalkley MJ, Drover MW, Peters JC. Catalytic N_2_–to–NH_3_ (or –N_2_H_4_) conversion by well–defined molecular coordination complexes. Chem Rev 2020; 120: 5582–636. 10.1021/acs.chemrev.9b00638 32352271 PMC7493999

[35] Roux Y, Duboc C, Gennari M. Molecular catalysts for N_2_ reduction: state of the art, mechanism, and challenges. Chem Phys Chem 2017; 18: 2606–17. 10.1002/cphc.201700665 28834039

[36] Ohki Y, Munakata K, Matsuoka Y et al. Nitrogen reduction by the Fe sites of synthetic [Mo_3_S_4_Fe] cubes. Nature 2022; 607: 86–90. 10.1038/s41586-022-04848-1 35794270

[37] Anderson JS, Rittle J, Peters JC. Catalytic conversion of nitrogen to ammonia by an iron model complex. Nature 2013; 501: 84–7. 10.1038/nature12435 24005414 PMC3882122

[38] Tanifuji K, Ohki Y. Metal–sulfur compounds in N_2_ reduction and nitrogenase–related chemistry. Chem Rev 2020; 120: 5194–251. 10.1021/acs.chemrev.9b00544 32459087

[39] Yang Q, Xu Q, Jiang HL. Metal–organic frameworks meet metal nanoparticles: synergistic effect for enhanced catalysis. Chem Soc Rev 2017; 46: 4774–808. 10.1039/C6CS00724D 28621344

[40] Wang HY, Cui L, Xie JZ et al. Functional coordination polymers based on redox–active tetrathiafulvalene and its derivatives. Coord Chem Rev 2017; 345: 342–61. 10.1016/j.ccr.2016.10.011

[41] Eizawa A, Arashiba K, Tanaka H et al. Remarkable catalytic activity of dinitrogen-bridged dimolybdenum complexes bearing NHC-based PCP-pincer ligands toward nitrogen fixation. Nat Commun 2017; 8: 14874–86. 10.1038/ncomms14874 28374835 PMC5382288

[42] Ashida Y, Mizushima T, Arashiba K et al. Catalytic production of ammonia from dinitrogen employing molybdenum complexes bearing N-heterocyclic carbene-based PCP-type pincer ligands. Nat Synth 2023; 2: 635–44. 10.1038/s44160-023-00292-9

[43] Čorić I, Mercado BQ, Bill E et al. Binding of dinitrogen to an iron–sulfur–carbon site. Nature 2015; 526: 96–9. 10.1038/nature15246 26416755 PMC4592811

[44] McSkimming A, Suess DLM. Dinitrogen binding and activation at a molybdenum–iron–sulfur cluster. Nat Chem 2021; 13: 666–70. 10.1038/s41557-021-00701-6 34045715

[45] Moula G, Nagasaki A, Matsumoto T et al. Synthesis of a nitrogenase PN-cluster model with [Fe_8_S_7_(μ-S_thiolate_)_2_] core from the all-ferric [Fe_4_S_4_(Sthiolate)_4_] cubane synthon. Angew Chem Int Ed 2021; 60: 15792–7. 10.1002/anie.202102369 33928749

[46] Li Z, Guo S, Sun Q et al. Electronic landscape of the P-cluster of nitrogenase as revealed through many-electron quantum wavefunction simulations. Nat Chem 2019; 11: 1026–33. 10.1038/s41557-019-0337-3 31570817

[47] Li Y, Li Y, Wang B et al. Ammonia formation by a thiolate-bridged diiron amide complex as a nitrogenase mimic. Nat Chem 2013; 5: 320–6. 10.1038/nchem.1594 23511421

[48] Wang XL, Yue W, Wang YX et al. Harnessing chromium(V) hydrazido intermediates for N_2_ functionalization to multisubstituted hydrazines through ligand migration. J Am Chem Soc 2025; 39: 35413–21. 10.1021/jacs.5c09083 40954099

[49] Xiong Y, Li B, Gu YM et al. Photocatalytic nitrogen fixation under ambient atmosphere using a porous coordination polymer with bridging dinitrogen anions. Nat Chem 2023; 15: 286–93. 10.1038/s41557-022-01088-8 36522581

[50] Hu HC, Cui P, Hu HS et al. Stable Zn^I^-containing MOFs with large [Zn_70_] nanocages from assembly of Zn^II^ ions and aromatic [Zn^I^_8_] clusters. Chem Eur J 2018; 24: 3683–8. 10.1002/chem.201705173 29230892

[51] Cao CS, Shi Y, Xu H et al. An uncommon multicentered Zn^I^–Zn^I^ bond-based MOF for CO_2_ fixation with aziridines/epoxides. Chem Commun 2021; 57: 7537–40. 10.1039/D1CC01865E 34236352

[52] Irene R, Ernesto C, Enrique G et al. Decamethyldizincocene, a stable compound of Zn(I) with a Zn-Zn bond. Science 2004; 305: 1136–8. 10.1126/science.1101356 15326350

[53] Li L, Li GD, Yan C et al. Efficient sunlight-driven dehydrogenative coupling of methane to ethane over a Zn^+^-modified zeolite. Angew Chem Int Ed 2011; 50: 8299–303. 10.1002/anie.201102320 21761525

